# Blood vessels as primary site of rejection in murine lung transplantation

**DOI:** 10.3389/ti.2026.16293

**Published:** 2026-05-29

**Authors:** Janne Kaes, Charlotte Hooft, Xin Jin, Tobias Heigl, Hengshuo Liu, Emilie Pollenus, Fran Prenen, Hanne Beeckmans, Pieterjan Kerckhof, Arno Vanstapel, Jan Van Slambrouck, Martina Mercurio, Marta Zapata-Ortega, Gitte Aerts, Vincent Geudens, Astrid Vermaut, Lise Vanvuchelen, Anse Somers, Lara Verbeylen, Cedric Vanluyten, Annalisa Barbarossa, Yousry Mohamady, Balin Özsoy, Andrea Zajacova, Birger Tielemans, Ali Onder Yildirim, Saskia Bos, Laurens De Sadeleer, Laurent Godinas, Joselyn Rojas Quintero, Francesca Polverino, Philippe E. Van den Steen, Laurens J. Ceulemans, Robin Vos, Bart M. Vanaudenaerde

**Affiliations:** 1 Laboratory of Respiratory Diseases and Thoracic Surgery (BREATHE), KU Leuven, Leuven, Belgium; 2 Institute of Lung Health and Immunity (LHI), Helmholtz Institute, Munich, Germany; 3 Laboratory of Immunoparasitology, Department of Microbiology, Immunology and Transplantation, Rega Institute for Medical Research, KU Leuven, Leuven, Belgium; 4 Translational Cell and Tissue Research, KU Leuven and UZ Gasthuisberg, Leuven, Belgium; 5 Department of Thoracic Surgery, University Hospitals Leuven, Leuven, Belgium; 6 Department of Respiratory Diseases, University Hospitals Leuven, Leuven, Belgium; 7 Pulmonary and Critical Care, Department of Medicine, Baylor College of Medicine, Houston, TX, United States

**Keywords:** blood vessels, chronic lung allograft dysfunction (CLAD), chronic rejection, flow cytometry, lung transplantation, murine orthotopic left lung transplantation, spatial proteomics

## Abstract

Survival after lung transplantation lags that of other solid organ transplants. Long-term survival is hampered primarily due to chronic lung allograft dysfunction (CLAD) development. It remains elusive how (chronic) rejection is organized within the lung graft over time post-transplant. Using a model of orthotopic left lung transplantation in major mismatched mouse strains with daily immunosuppression, we aimed to study the spatiotemporal dynamics of (chronic) rejection, using micro-computed tomography imaging, flow cytometric analyses and spatial proteomics. Endothelial cells demonstrated early activation and destruction (day 7 post-transplant). The accompanying early inflammation at the vascular compartment, progressed towards aberrant tissue repair resulting in irreversible bronchovascular fibrosis and chronic graft dysfunction. We provide new insights in the spatiotemporal dynamics of (chronic) rejection with a vascular-oriented onset that may have future implications for diagnosis and treatment in clinical lung transplantation.

## Graphical Abstract

**Figure:**
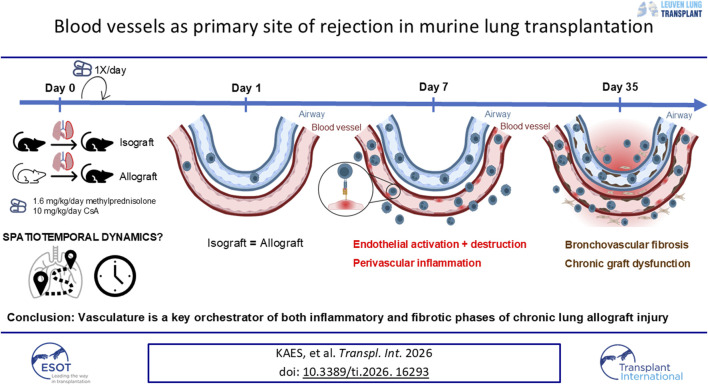

## Introduction

Lung transplantation is the only curative treatment for end-stage lung diseases. Despite advances in donor selection, surgical techniques, and immunosuppressive strategies, survival after lung transplantation lags that of other solid organ transplants with a median survival of only 6 years post-transplantation [1–3]. Beyond the first post-transplant year, survival is predominantly limited by the development of chronic lung allograft dysfunction (CLAD) [1, 4, 5]. CLAD is clinically defined by an irreversible decline in lung function and represents an umbrella term encompassing heterogeneous pathological processes. It is not exclusively defined by rejection, but rather as a broad term for irreversible loss of lung function. Three clinical phenotypes of CLAD are currently recognized, including, ‘bronchiolitis obliterans syndrome’ (BOS), ‘restrictive allograft syndrome’ (RAS) and a mixed phenotype [6]. These phenotypes reflect the complexity of CLAD from a clinical point of view. Importantly, this CLAD definition does not provide insights into the underlying drivers of graft injury [1, 5]. CLAD has historically been conceptualized as a predominantly airway-centered disease, with clinical and research efforts largely focused on bronchiolar pathology [7–9]. However, this airway-centric view contrasts with established paradigms of vascular chronic rejection that is well characterized in other solid organ transplants, where chronic rejection has been perceived as a vessel-centered process in which persistent immunological and endothelial activation induces thrombosis, intimal proliferation, and vascular remodeling act as hallmarks of chronic allograft vasculopathy [10–12].

The apparent disconnect between lung transplantation and other solid organs is therefore striking, particularly given the shared immunological principles governing allograft rejection. Considering transplant rejection can be described as an immune-mediated recognition of the donor graft HLA antigens as ‘non-self’, where both cellular and humoral mechanisms of the innate and adaptive immune system get involved [13].

So, despite vascular chronic rejection being well characterized in other transplanted organs and some studies within the field of lung transplantation reporting perivascular inflammation histologically, its central role and the temporal and spatial relationships between vascular injury and graft dysfunction have never been investigated in the context of lung transplantation, and direct evidence in human lung allografts remains limited [9, 11, 12, 14]. This conceptual gap has contributed to an incomplete understanding of chronic lung allograft injury and may partially explain the lack of effective targeted therapies [1, 15, 16].

Importantly, the traditional dichotomous classification of rejection into “acute” and “chronic” forms complicates interpretation. In clinical practice, rejection cannot be regarded as a binary or purely temporal process. Lung transplant recipients may experience chronically ongoing antibody-mediated rejection, recurrent or persistent episodes of acute rejection, or overlapping immunopathological processes resulting in chronic graft injury [17]. Distinguishing “chronic” rejection as a pathophysiological mechanism from chronically ongoing forms of “acute” rejection is therefore essential [12, 18–20]. Controlled experimental models may act as a valuable tool to obtain essential and fundamental information on the spatiotemporal dynamics of vascular involvement in (chronic) rejection [21–24].

Building on these observations, we hypothesize that (chronic) rejection in lung transplantation may originate in the vascular compartment with a significant role for endothelial cells. We aim to map the temporal and spatial progression of vascular pathology in a murine lung transplant model to understand how vascular injury contributes to chronic graft dysfunction. We will use our murine model of rejection after orthotopic left lung transplantation and use advanced methodologies including multicolor flow cytometry, *in vivo* and *ex vivo* µCT imaging and spatial proteomics.

## Materials and methods

### Orthotopic mouse left lung transplantation and study design

**FIGURE 1:**
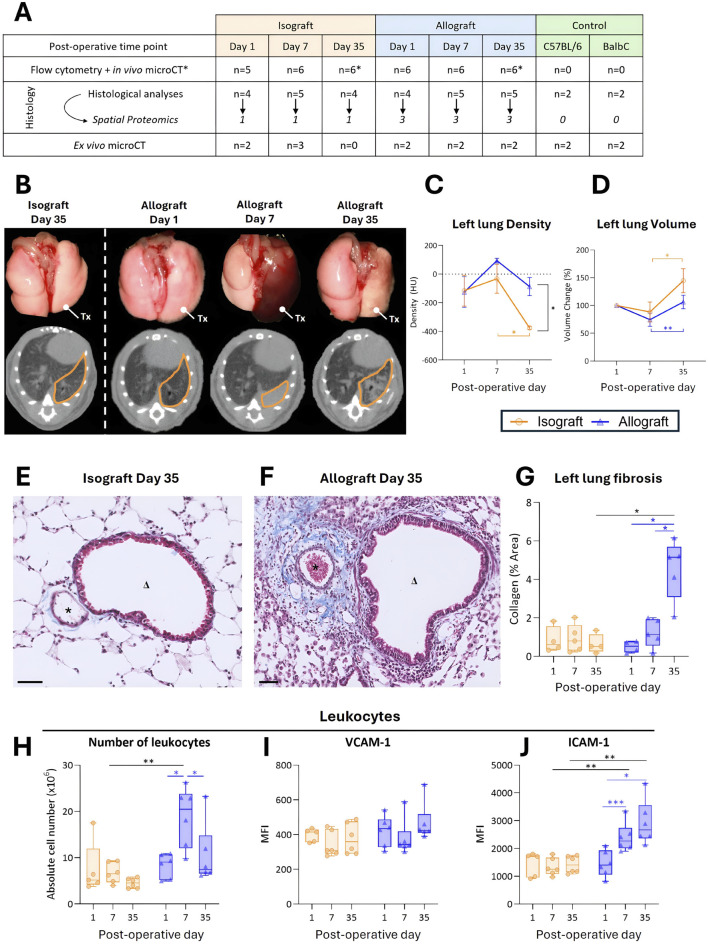
Study design and standard elements of the murine orthotopic left lung transplantation. **(A)** Orthotopic left lung transplantation was performed to create isografts (C57BL/6 NRj in C57BL/6 NRj) and allografts (Balb/cJRj in C57BL/6 NRj). *In vivo* µCT was performed on the same groups of mice used for flow cytometry sacrificed on day 35. Animals used for histology were also used to perform spatial proteomics. **(B)** Representative macroscopic pictures of lung blocs with the corresponding µCT images. The left transplanted lung is indicated with Tx and delineated in yellow on µCT sections. **(C)** Mean lung density difference in left transplanted lung, calculated from end-expiratory µCT images. Mean is shown with error bars indicating SEM. **(D)** Evolution of total lung volume of left transplanted lung with relative change compared to the measurement of day one. Volumes were calculated based on end-expiratory µCT images. Data is expressed as mean with SEM. **(E,F)** Masson’s trichrome images of isograft and allograft on day 35 after transplantation, collagen deposition is indicated in blue. Scale indicates 50 μm; * = blood vessel; **Δ** = airway. **(G)** Collagen staining intensity was quantitatively calculated in tissue sections stained with Masson’s trichrome using QuPath software. **(H)** Flow cytometry was used to determine absolute numbers of leukocyte cells and to determine the surface expression of adhesion molecules on these leukocytes **(I)** ICAM-1 and **(J)** VCAM-1. Data are shown as box-and-whisker plots (box: median with interquartile range, whiskers: full data distribution) with each dot representing an individual mouse sample. Tx = transplanted; HU = Hounsfield units; ISO = isograft; ALLO = allograft; MFI = mean fluorescence intensity.

Mice (male) were purchased from Janvier Labs (Le Genest-Saint-Isle, France) and transplanted between eight to 10 weeks of age. Recipients were C57BL/6N (H-2K^b^) and donors were either C57BL/6N (H-2K^b^) for isograft transplantation or BalbC (H-2K^d^) for allograft transplantation. Serial sacrifice was performed at three different time points, on post-operative day 1, 7 and 35. All mice were housed in a conventional facility with individually ventilated cages (IVC) and received *ad libitum* standard chow and water. Orthotopic left lung transplantation was performed as previously described [22, 25, 26]. After surgery, mice were allowed to recover on a heating pad overnight and received buprenorphine for the following 72 h. Both isografts and allografts received daily subcutaneous immunosuppression consisting of 10 mg/kg/day cyclosporine (Sandimmun®, Novartis, Vilvoorde, Belgium) and 1.6 mg/kg/day methylprednisolone (SoluMedrol®, Pfizer, Brussels, Belgium) started immediately after transplantation. The specifics of the study design are illustrated in Figure 1A. The experimental procedure was approved by the Ethical Committee for Animal Research at KU Leuven (P194/2019). To determine leukocyte and endothelial cell numbers and activation, flow cytometry was performed on the entire left lung graft at the three timepoints (n = 5–6/group/timepoint). Longitudinal, non-terminal *in vivo* µCT imaging was performed on day 1, 7 and 35 in the grafts (n = 6/group) of the flow cytometry experiment sacrificed day 35. Terminal exsanguination with retro-orbital blood collection was performed, prior to sacrifice for measuring the cyclosporine A trough level by an immunoassay (Dimension® RXL, Siemens Medical solutions, Diamond diagnostics, USA) (Supplementary Figure S1). The experiment with isografts and allografts was repeated for histological analyses (n = 4–5/group/timepoint). At the time of sacrifice, lungs were perfused with saline and inflated, followed by perfusion with 4% PFA at 4 °C for 24 h. The grafts were subsequently embedded in paraffine and processed into 4 µm thick sections which were stained with Hematoxylin-Eosin and Masson’s trichrome. Images were taken with an Olympus BX61 microscope. The percentage of collagen in a stained lung section with Masson’s trichrome was quantified using QuPath 0.3.2 with a personalized Pixel Classifier created with the ‘Train Pixel Classifier’ tool.

### Flow cytometric analysis to quantify leukocyte and endothelial cell numbers and activation

At time of autopsy, the transplanted lung was removed and cells were isolated as previously described [27]. The lung was extracted and collected in RPMI buffer [RPMI GlutaMAX/FCS (5%)/ 1% penicillin/streptomycin/ 0.1% beta-mercaptoethanol], minced and incubated for 30 min at 37 °C in digestion medium consisting of 2 mg/mL collagenase D and 0.1 mg/mL DNase I in RPMI buffer. Subsequently, lung tissue was homogenized using a 20-gauge needle and new digestion medium was added, followed by a second incubation period of 15 min at 37 °C. Cells were washed and red blood cell lysed using 0.83% NH_4_Cl/10 mM Tris at 37 °C and passed through a 70 µm nylon cell strainer. After a last washing step, live leukocytes were counted in a Bürker chamber with trypan-blue staining.

For the flow cytometry 1.5-3 million lung cells were used. Cells were incubated with a viability dye, Zombie Aqua (Biolegend, San Diego, CA, USA) or Zombie UV (Biolegend), together with Mouse Fc block (MACS Miltenyi Biotec.) for 15 min at room temperature. After washing twice, cells were stained with a panel of monoclonal antibodies (Supplementary Table S1) dissolved in Brilliant stain buffer (BD Biosciences, Erembodegem, Belgium) for 20 min at 4 °C. After surface staining, 100,000 or 200,000 live single cells were analyzed per sample with a BD LSR Fortessa Flow cytometer (BD Biosciences). The flow cytometric panels used allow to quantify viable cells, leukocytes, endothelial cells numbers and activation status (Supplementary Table S1). For the activation status we used MHC1/2 (central for T cell activation), adhesion molecules ICAM-1 (present on leukocytes and endothelial cells) and VCAM-1 (present on endothelial cells). Data were analyzed with FlowJo v10 software (FlowJo LLC, Ashland, OR, USA) and cells were gated according to predefined gating strategies (Supplementary Figure S2). The absolute number of leukocytes and endothelial cells were calculated by the percentage of CD45^+^ and CD45^−^ CD31^+^ cells, respectively, among the live cells multiplied by the total number of live cells determined with the Bürker chamber.

### *In vivo* and *ex vivo* µCT imaging to identify and locate rejection

For *in vivo* µCT imaging free-breathing mice were scanned with a whole-body small animal µCT scanner (SkyScan 1278, Bruker µCT, Kontich, Belgium). Animals were anesthetized with isoflurane (2% in pure oxygen) and placed in supine position. Following scan parameters were used: 50 kVp X-ray source, 350 µA current, 1 mm aluminum X-ray filter and 150 ms exposure time per projection acquiring projections with 0.9° increments over a total angle of 220°. Acquisition resulted in a respiratory-weighted reconstructed 3D dataset with an isotropic voxel size of 50 µm in a total scan time of 3 min, associated with a radiation dose exposure of 69–89 mGy [28]. The images were reconstructed with the following parameters: smoothing of one, beam-hardening correction of 10%, post-alignment and ring artefact reduction were optimally set for each individual scan. Images were processed and calibrated to Hounsfield units (HU) as described before [29]. Image reconstruction, analysis and quantification were performed using NRecon, DataViewer and CTAn software provided by the manufacturer. Quantification of total lung volume and mean lung density was performed for a manually delineated region of interest (ROI), resulting in a volume of interest (VOI) on the transversal µCT images at end-expiration.

For *ex vivo* µCT imaging separate transplant allograft, isograft and non-transplant control mice were used (Figure 1A). The lungs were fixated as previously described and chemically dehydrated in a graded series of ethanol concentrations followed by hexamethyldisilazane (Sigma-Aldrich, Overijse, Belgium) submersion overnight. Dried lungs were scanned using a Skyscan 1272 µCT scanner (Bruker, Kontich, Belgium) with a resolution of 3.5 µm. 3D images were reconstructed using the NRecon software (version 1.7.0.4, Bruker microCT, Kontich, Belgium) and 3D segmentations of airway lumina, arteries and veins was performed semi-automatically by ITK-SNAP software [30].

### Bulk spatial GeoMx proteomics to validate vessel orientated rejection in lung sections

GeoMX spatial proteomic profiling was performed to determine the spatial localization of different immune-related proteins of interest. By selecting specific regions of interest based on the anatomical location, we performed a regional analysis subdividing airways, arteries, veins, and parenchyma (Figures 5A,B). Formalin-fixed and paraffin-embedded (FFPE) lung tissue sections (4 µm) were obtained from one isograft at each time point (day 1, 7 and 35) and three allografts at each timepoint. For each tissue section, three regions of interest (ROIs) were selected and paired for the parenchyma, bronchioles, arteries, and veins (total of 12 ROIs), based on the anatomical orientation in the H&E image. For the spatial proteomics, the GeoMx assay and platform was performed at the F Polverino lab in Baylor (Houston, USA). Tissue sections were incubated with 47 oligo-labelled primary antibodies of interest (Supplementary Table S2) and analyzed using the Nanostring GeoMx Digital Spatial Profiler as per manufacturer’s instructions. The detection antibodies comprised three fixed panels of the GeoMx assay (GeoMx immune cell profiling panel, GeoMx immune activation status panel, GeoMx immune cell typing panel).

### Statistical analysis

Data analysis was performed using GraphPad statistical software (Prism, version 10, San Diego, CA, USA). To compare the different groups one-way ANOVA was used. To compare the different time points in isografts or allografts, a mixed effects model with Šidák multiple comparisons test was conducted. To compare isografts and allografts, a mixed effect model with Tukey’s multiple comparisons *post hoc* was used. A p-value of <0.05 was considered significant. Differential protein expression analyses (spatial proteomics) were performed to compare allografts and isografts in different compartments of the lung. Third quartile normalization was performed on the obtained expression matrix to allow comparison between samples. Of the 47 included proteins, 35 were assigned to a specific cluster (structural, hematopoietic, innate immunity, adaptive immunity, stromal and proliferation). To be able to pool multiple proteins per cluster, expression values were scaled to Z-scores for each protein separately. Statistical analysis on these differences was performed using mixed modeling in R (version 4.2.2), using the “lmer” function. The model used a full factorial design between graft (isograft and allograft), day (1, 7, 35) and tissue type (airway, artery, vein, parenchyma), with notation “graft*day*tissue”. Different mouse individuals were included as random effect. The least squares method (“emmeans” function) was used to perform pairwise comparisons between allograft and isograft.

## Results

### Lung morphology and density after murine orthotopic single lung transplantation

Isografts maintained normal macroscopic appearance throughout follow-up, whereas allografts progressively developed yellow discoloration with patchy dark areas by day 35 (Figure 1B). Longitudinal *in vivo* µCT revealed normal morphology in both groups on day 1; by day 7, allografts showed complete consolidation corresponding to atelectasis, while isografts remained aerated. On day 35, patchy dense regions persisted in allografts, contrasting with preserved isograft structure. Quantitative analysis confirmed these changes: mean lung density was significantly higher in allografts compared to isografts on day 35 (−87 ± 154 HU vs. −376 ± 27 HU, p = 0.016), while isografts showed a modest decline in density over time (p = 0.043) (Figure 1C). Lung volumes increased from day 7 to 35 in both groups (isograft p = 0.04; allograft p = 0.008) with no overall differences between both groups (Figure 1D). Histology confirmed these findings by preserved parenchymal architecture without cellular infiltrates or connective tissue deposition and fibrosis formation in isografts versus bronchovascular inflammation and fibrosis in allografts on day 35 (Figures 1E–G; Supplementary Figure S3).

### Flow cytometry for leukocyte and endothelial cell numbers and activation status

The number of leukocytes (CD45^+^) significantly increased in the allograft left lung on day 7 (p = 0.037) but decreased again on day 35 (p = 0.020) (Figure 1H). These leukocytes significantly presented more ICAM-1 on their surface on day 7 (p = 0.0026) and 35 (p = 0.0045) compared to isografts, while no change in VCAM-1 was observed (Figures 1I,J).

**FIGURE 2:**
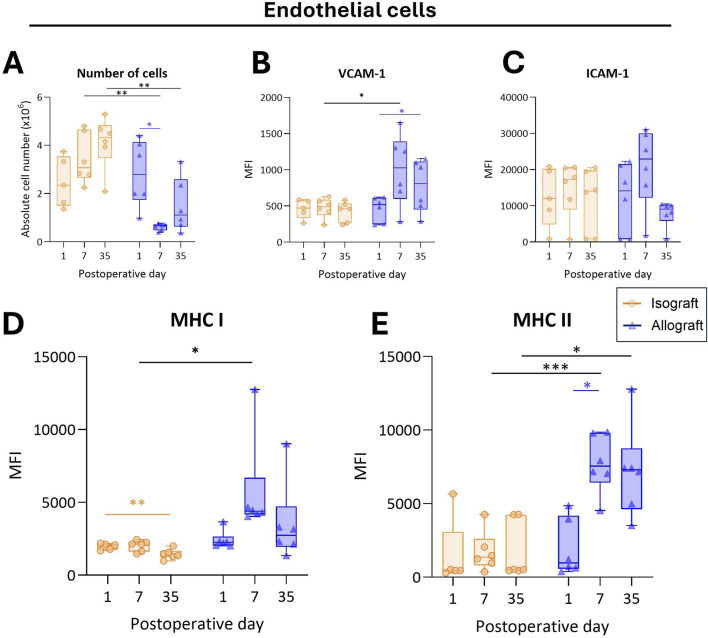
Total endothelial cell numbers and endothelial surface expression of adhesion and MHC molecules in the murine left transplanted lung. Isograft (C57BL/6NRj in C57BL/6NRj) and allograft (Balb/cJRj in C57BL/6NRj) transplantations were performed and sacrificed at indicated time points. **(A)** Absolute number of CD31^+^ endothelial cells. **(B–E)** Flow cytometry was used to determine the surface expression of adhesion molecules (VCAM-1 and ICAM-1) and MHC molecules (MHC I and II) on CD31^+^ endothelial cells. Data are shown as box-and-whisker plots (box: median with interquartile range, whiskers: full data distribution), with each dot representing an individual mouse sample. EC = endothelial cells; Leuko = leukocytes; MFI = mean fluorescence intensity.

Endothelial cell (CD31^+^) numbers remained stable in isografts. In contrast, allografts showed a significant reduction on day 7 compared to day 1 (p = 0.024), which only partially recovered by day 35 (p = 0.199) (Figure 2A). Endothelial cells showed to be activated, as VCAM-1 was significantly increased on day 7 compared to isografts (p = 0.047) and remained increased on day 35 versus day 1 (p = 0.049) (Figure 2B). No change in ICAM-1 expression by endothelial cells was observed (Figure 2C). Endothelial MHCI and MHCII expression were increased in allografts compared to isografts on day 7 (p = 0.045; p = 0.0002, respectively) and MHCII remained significantly increased on day 35 (p = 0.006) (Figures 2D,E).

### Histological visualization of vessel-centered rejection

**FIGURE 3:**
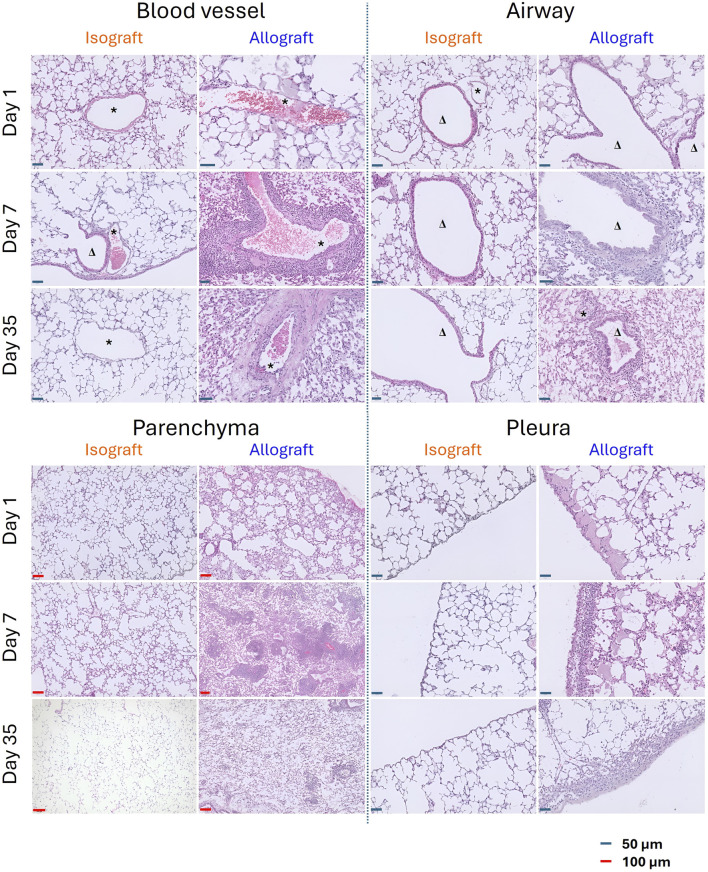
Representative histological images of isografts and allografts of the different compartments (blood vessel, airway, parenchyma, and pleura) over time. Representative Hematoxylin-Eosin (H&E) staining of the different groups on day 1, 7, and 35 after transplantation (n = 4/5). Scale bar of parenchymal images indicates 100 μm, scale bar of all other images indicates 50 µm, * = blood vessel; **Δ** = airway.

Isografts showed preserved vascular, airway, and pleural architecture, with minimal focal edema on day 1 that resolved by day 35. Allografts displayed a vascular driven pathology. On day 1, the multifocal intra-alveolar edema was accompanied by subtle perivascular changes (Figure 3). By day 7, dense cuff-like perivascular infiltrates dominated, composed of lymphocytes and histiocytes, frequently accompanied by pleural extension. On day 35, these infiltrates became organized and shifted in composition toward plasma cell–rich cuffs with fewer lymphocytes, paralleled by progressive perivascular fibrosis (Figure 3; Supplementary Figure S3). The parenchyma showed edema on day 1 that was largely resolved in isografts but more extensive in allografts (Figure 3). The airways remained preserved in isografts, whereas allografts demonstrated peribronchial fibrosis on day 35 (Figure 3; Supplementary Figure S3).

### Bulk spatial GeoMx proteomics confirm vessel orientated rejection

**FIGURE 4:**
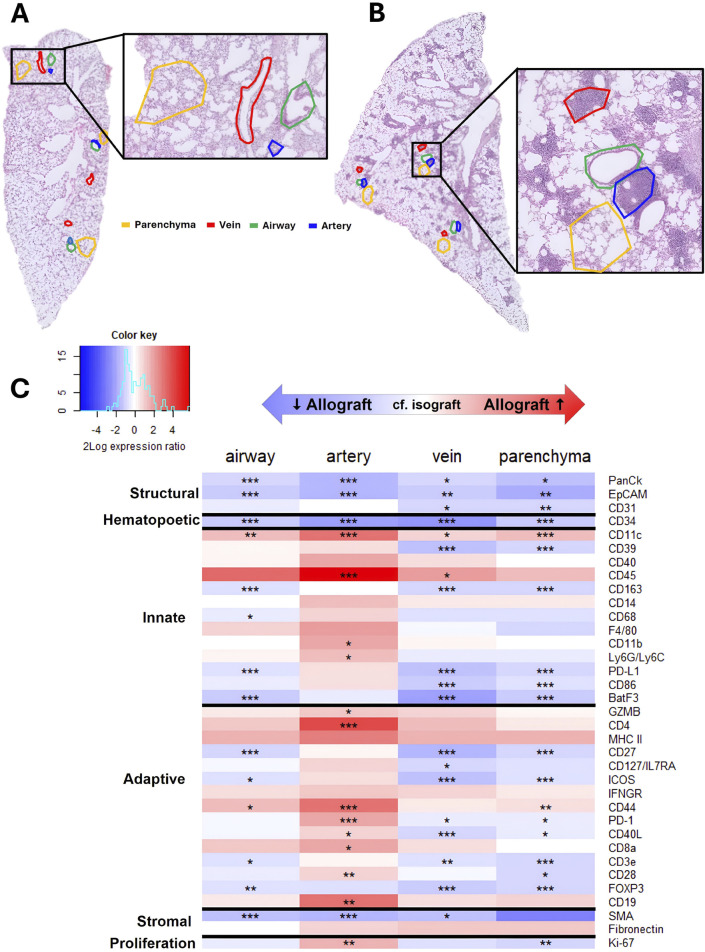
Spatial proteome analysis at regions of interest: artery, vein, airway, and parenchyma. **(A,B)** Example of allograft on day 1 and day 35 after transplantation with regions of interest (ROIs), yellow indicates parenchyma, red vein, green airway, and blue artery. **(C)** Heatmap of spatial proteomics according to the anatomical location of the immune process in the transplanted lung. Protein expression levels were quantified around the airways, arteries, veins and in the parenchyma region of interest in formalin-fixed paraffin embedded (FFPE) tissue sections from the left transplanted lungs of isografts (C57BL/6NRj in C57BL/6NRj) (n = 1/time point) and allografts (Balb/cJRj in C57BL/6NRj) (n = 3/time point) by NanoString GeoMX digital spatial profiling. On day 1, no significant differences between allograft and isograft were observed. Data of day 1 was discarded for this figure (and excluded from the model). Additionally, since no major differences were observed between day 7 and day 35, data of day 7 and 35 were pooled together for simplicity of the figure. This was performed by still including day 7 and 35 in the model but only performing pairwise comparisons between allograft and isograft for all tissue regions. Z-score differences between allograft and isograft were hence computed for each cluster and tissue region separately (colors). The mixed model provided p-values of the pairwise comparisons are indicated on the figure.

2D-UMAP projection of all included samples, based on normalized protein expression, showed isografts at all timepoints clustering together with allograft day 1, while a second cluster consisted of allograft day 7 and 35 together (Supplementary Figure S4). Indeed, no significant differences were found comparing isografts and allografts on day 1. To continue, based on this clustering, data of allograft day 7 and 35 were pooled and this was compared to isograft (Figure 4C). We observed a higher expression of most of the proteins at the arterial regions in allografts compared to isografts. Particularly CD45 (pan leukocyte marker), CD4 and CD44 (T cell markers) were more present around the arteries of allografts compared to isografts (p < 0.001; p = 0.001; p = 0.002, respectively) and the other structures. Expression of CD19 (B cell marker) was also higher around the arteries of allografts compared to isografts (p = 0.006). Both innate and adaptive immune markers were more expressed in the allografts’ arteries compared to isografts and other structures. The expression of CD11c (dendritic cell marker) was increased in allografts in the parenchyma (p = 0.001) and around the arteries (p = 0.006) and airways (p = 0.029), compared to isografts. Protein expression of immune regulatory markers CD39, CD163, PD-L1 and FoxP3 decreased in the allograft’s parenchyma (p < 0.001; p < 0.001; p < 0.001; p < 0.001) and veins (p < 0.001; p < 0.001; p < 0.001; p = 0.002) compared to isografts. Expression of other adaptive immunity markers CD27, ICOS and CD40L (T cell activation markers) were significantly less detected in allografts’ vein compared to isografts (p < 0.001; p < 0.001; p < 0.001). EpCAM and PanCK (epithelial markers) were decreased in allografts’ airways (p < 0.001; p < 0.001) in comparison to isografts. Smooth muscle actin (SMA) was significantly lower in allografts’ airways (p < 0.001) and arteries (p < 0.001). However, fibronectin seemed to be higher in allografts, although not significant. Ki-67 (proliferation marker) was expressed more highly around the arteries and parenchyma of the allografts compared to the other structures, and to isografts (p = 0.023; p = 0.023).

### 3D visualization by *ex vivo* µCT imaging to locate rejection

**FIGURE 5:**
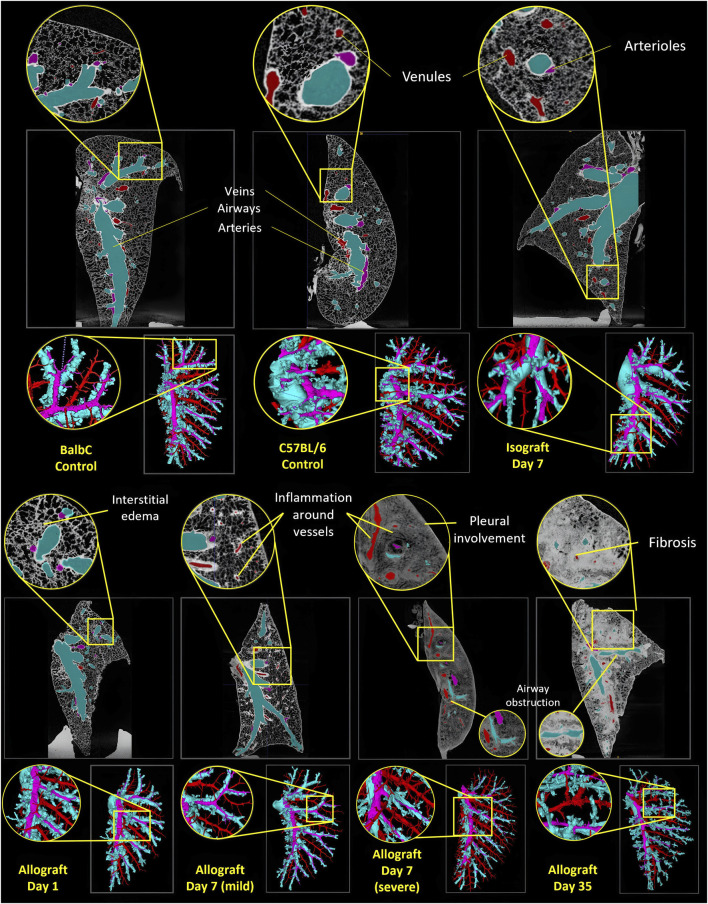
Representative *ex vivo* high resolution µCT cross-sections of the left (transplanted) lung with 3D segmentation. Allografts (Balb/cJRj in C57BL/6NRj) left transplanted lung at three different time points after murine single lung transplantation. Left lung of a non-transplant BalbC mouse for comparison, showing healthy parenchyma with open airways, lined by an artery. Allograft on day 1 showing interstitial edema. On day 7, an increased density was noticed around the blood vessels, this might indicate perivascular inflammation. Allograft on day 35 after transplantation showed intense increased density in the whole lung, which could be pulmonary fibrosis. 3D segmentation shows a prominent decrease in blood vessel density and branching in transplanted mice compared to control. Blue indicates airways, purple indicates arteries and red shows the veins.

*Ex vivo* µCT was performed on donor controls, isografts, and allografts on day 1, 7, and 35, enabled segmentation of the airway tree, pulmonary arteries, and veins (Figure 5). Donor control lungs (Balb/c and C57BL/6) and isografts demonstrated preserved morphology of both airway and vascular compartments at all time points. Allografts appeared normal on day 1, with intact airways and vasculature comparable to controls. In contrast, by day 35, allografts exhibited marked vascular pathology: arteries and veins were narrowed, collapsed, and embedded within dense fibrotic regions. Notably, airway structures appeared intact without evidence of distortion or malformation and airway obstruction was only found secondary and in severe stages of rejection.

## Discussion

This study demonstrated a vasculature and endothelial cell centered onset of rejection in a mouse model of chronic lung rejection. Our findings indicate that endothelial cells are key in the immune response of acute rejection (day 7) and the fibrotic remodeling of chronic rejection (day 35) around the arteries, analogous to chronic vascular injury observed in other solid organ transplants.

We previously characterized the alloimmune response in this mouse transplant model with both innate and adaptive immune cells involved [22, 25]. In this study we confirmed the temporal relation between immune activation on day 7 (with little fibrosis), and organized fibrosis on day 35 in the allografts. As such, day 7 may be regarded as acute rejection and day 35 may be regarded as chronic rejection. In the current study, we observed an early (day 7) loss of endothelial cells that coincided with this inflammatory peak of the acute phase of rejection. Moreover, early lymphocyte activation and leukocyte adhesion to the endothelium was enhanced by endothelial cell activation with increased expression of VCAM-1 and increased expression of ICAM-1 in leukocytes. The parallel increase in MHC molecules on endothelial cells (especially MHCII) further supports the leading role for the endothelium in orchestrating the immune response, by activating the adaptive immune system with its antigen-presenting capacity, directly activating T-helper cells. Jambusaria et al. demonstrated that lung endothelial cells express higher levels of immunoregulatory genes than those from the brain or heart, underscoring their unique immune-modulating capacity [31]. Carmeliet et al. further introduced the concept of “immunomodulatory endothelial cells” (IMECs), a specialized subset capable of engaging directly in immune signaling [32]. Histology and especially the spatial proteomics highlighted compartment-specific patterns of injury, confirming innate and adaptive organization specifically localized to arteries. Our findings suggest that endothelial cells function not only as targets of the immune response but also as active contributors to ongoing alloimmune activation within the graft. Since the alloantigenic stimulus persists, the immune response cannot resolve. Continuous endothelial activation and injury likely promote maladaptive repair processes, further resulting in perivascular fibrosis. This remodeling extends into adjacent parenchymal, pleural and airway compartments, providing a mechanistic link between early vascular injury and later graft dysfunction. Collectively, these observations position the vasculature as a central orchestrator of both the inflammatory and fibrotic phases of chronic lung allograft injury.

How this concept of a vessel-centered rejection process translates to the human clinical setting with CLAD (both RAS and BOS) is highly relevant. Acute rejection is histologically confirmed by the presence of lymphocytic perivascular infiltrates in the clinic, which is in parallel with the acute rejection observed on day 7 in the mice. In addition, some human studies highlighted the vascular contribution to CLAD, with reduced VEGF levels in bronchoalveolar lavage fluid of patients with RAS [9], and vascular remodeling, including pulmonary arteriopathy and venopathy, has been described in both BOS and RAS [7, 33].

To extend our molecular and histological observations to the macrostructural level, a high-resolution *ex vivo* microCT was used enabling segmentation of the airway tree and pulmonary vascular compartments. Consistent with our molecular findings, vascular structures in allografts showed progressive narrowing, pruning, and remodeling by day 35, while airway architecture remained largely preserved, providing 3D confirmation that chronic graft injury in this mouse model primarily affects the vasculature rather than the airway compartment. This vascular involvement suggests that vascular injury precedes or drives subsequent parenchymal or airway remodeling which ultimately leads to loss of lung function. From a translational perspective, this also implies that, potentially, *in vivo* imaging approaches focusing on vascular alterations could serve as sensitive tools to detect CLAD progression in clinical lung transplantation at earlier stages, prior to the decrease in lung function. Advanced imaging modalities capable of assessing pulmonary perfusion or vascular integrity may therefore hold diagnostic value in identifying patients at risk for CLAD. Moreover, therapeutic strategies aimed at preserving endothelial integrity or modulating vascular inflammation might complement current immunosuppressive regimens and offer a new avenue to improve long-term graft survival.

The limitations of this study are the nature of the model, being a highly controlled setting to dissect alloimmune mechanisms, which cannot fully replicate the complexity of human lung transplantation. Immunosuppressive regimens also differ between species; we used cyclosporine A to moderate the immune response, whereas tacrolimus-based therapy is standard in clinical practice, which may affect the kinetics and nature of rejection. Only male mice were included, and potential sex-specific immune differences therefore remain unexplored. Furthermore, the number of isograft controls was limited, which may reduce statistical power for some comparisons. Mechanistic pathways underlying endothelial cell loss and its direct effects on fibroblast activation and fibrosis were not addressed here but represent an important direction for future work. Finally, this is a murine model of rejection, not CLAD. Modeling CLAD requires graft-specific functional measurements, and their absence is a limitation of this study. Despite these limitations, this model and study offered a unique opportunity to study rejection processes under reproducible and isolated conditions, minimizing confounding factors that are difficult to control in human transplantation.

In conclusion, the current study reveals that chronic vascular injury is a central feature of rejection also in the lung, preceding and potentially driving fibrotic remodeling. By identifying the endothelium as an active participant rather than a passive target, this study redefines lung rejection as a vessel-centered process. Targeting vascular inflammation and preserving endothelial integrity may thus hold the key to improving long-term lung allograft survival. This study invites to investigate how vascular rejection fits into the concept of CLAD.

## Data Availability

The raw data supporting the conclusions of this article will be made available by the authors, without undue reservation.
