# Supplementary material for: Blood vessels as primary site of rejection in murine lung transplantation
Source: Transpl Int. 2026 May 29;39:16293. doi: 10.3389/ti.2026.16293 (PMC13260051; doi:10.3389/ti.2026.16293)
Supplement: Supplementary file 1 [file Supplementaryfile1.docx]

**Supplementary Figure S1: Cyclosporine A trough levels in blood**. No differences in serum cyclosporine A levels were observed between the groups: isografts versus allografts, nor within these groups over time.

**Supplementary Figure S2: Gating strategy for pulmonary leukocytes and pulmonary endothelial cells with expression of adhesion and MHC molecules.** Lung cells were isolated and stained for flow cytometry. After exclusion of debris and cell doublets, all live cells (LD) were gated. Endothelial cells were identified by CD31 staining. Leukocytes were identified by CD45 staining. Alveolar macrophages were excluded by plotting Live/Dead versus FSC-A as these cells are autofluorescent and result in false positive staining for lymphoid cell markers.

**Supplementary Figure S3: Representative histological images of whole lung slices of isografts and allografts over time.** Representative Masson trichrome staining of the different groups on day 7 and 35 after transplantation. Blue staining shows collagen deposition. Scale bar indicates 800 µm. _*_ **=** blood vessel; **Δ** = airway.

**Supplementary Figure S4: Spatial proteomics.** 2D**-**UMAP with all included samples shows the clustering of allografts on day 7 and allografts on day 35.
