# Supplementary figures and images for: Blood vessels as primary site of rejection in murine lung transplantation
Source: Transpl Int. 2026 May 29;39:16293. doi: 10.3389/ti.2026.16293 (PMC13260051; doi:10.3389/ti.2026.16293)

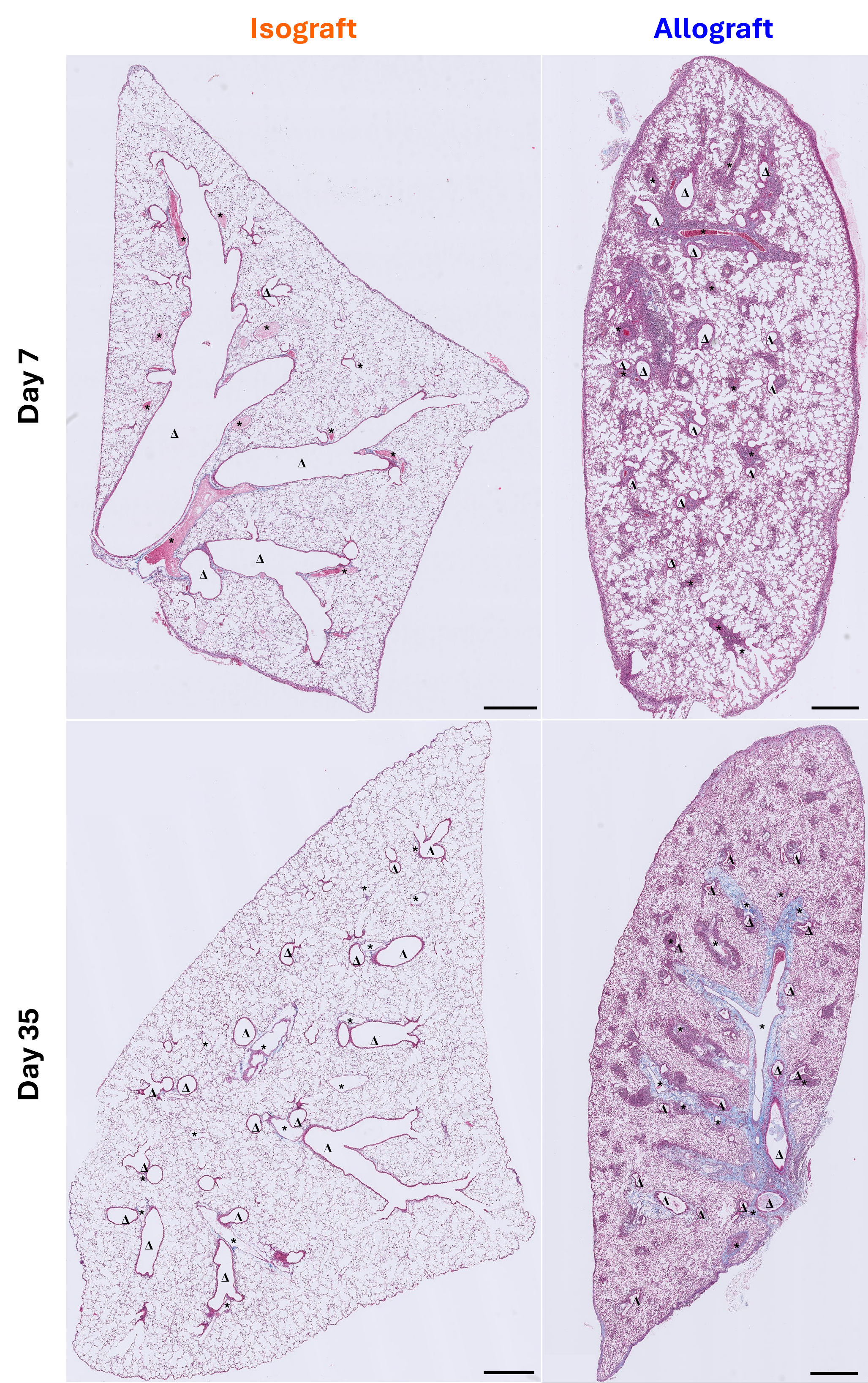

Supplement: Supplementary file 2 [file Image3.tif]

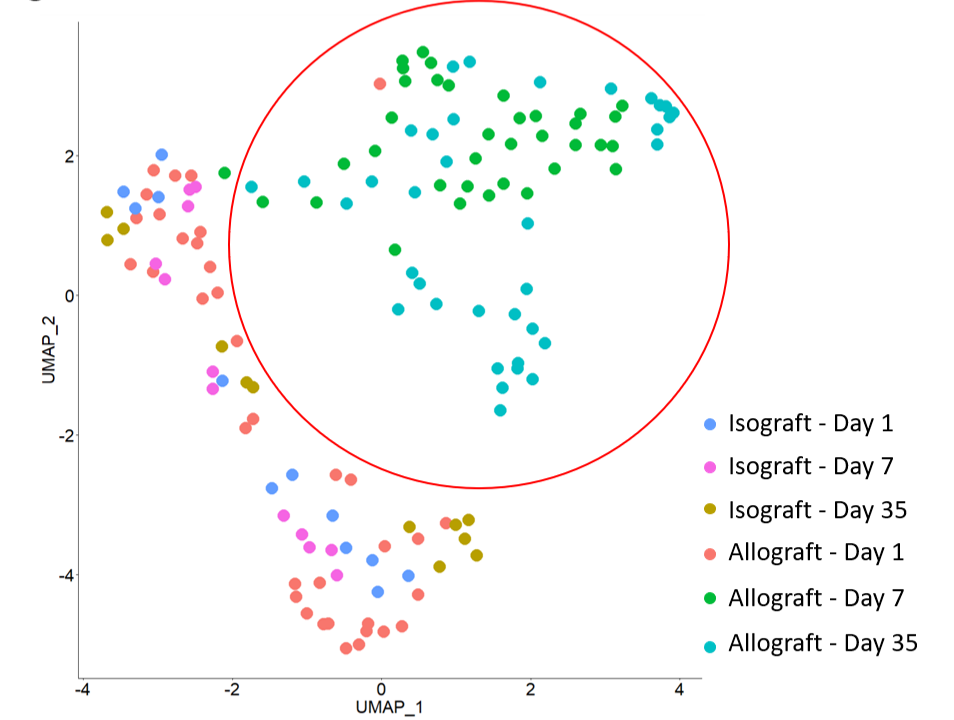

Supplement: Supplementary file 3 [file Image4.tif]

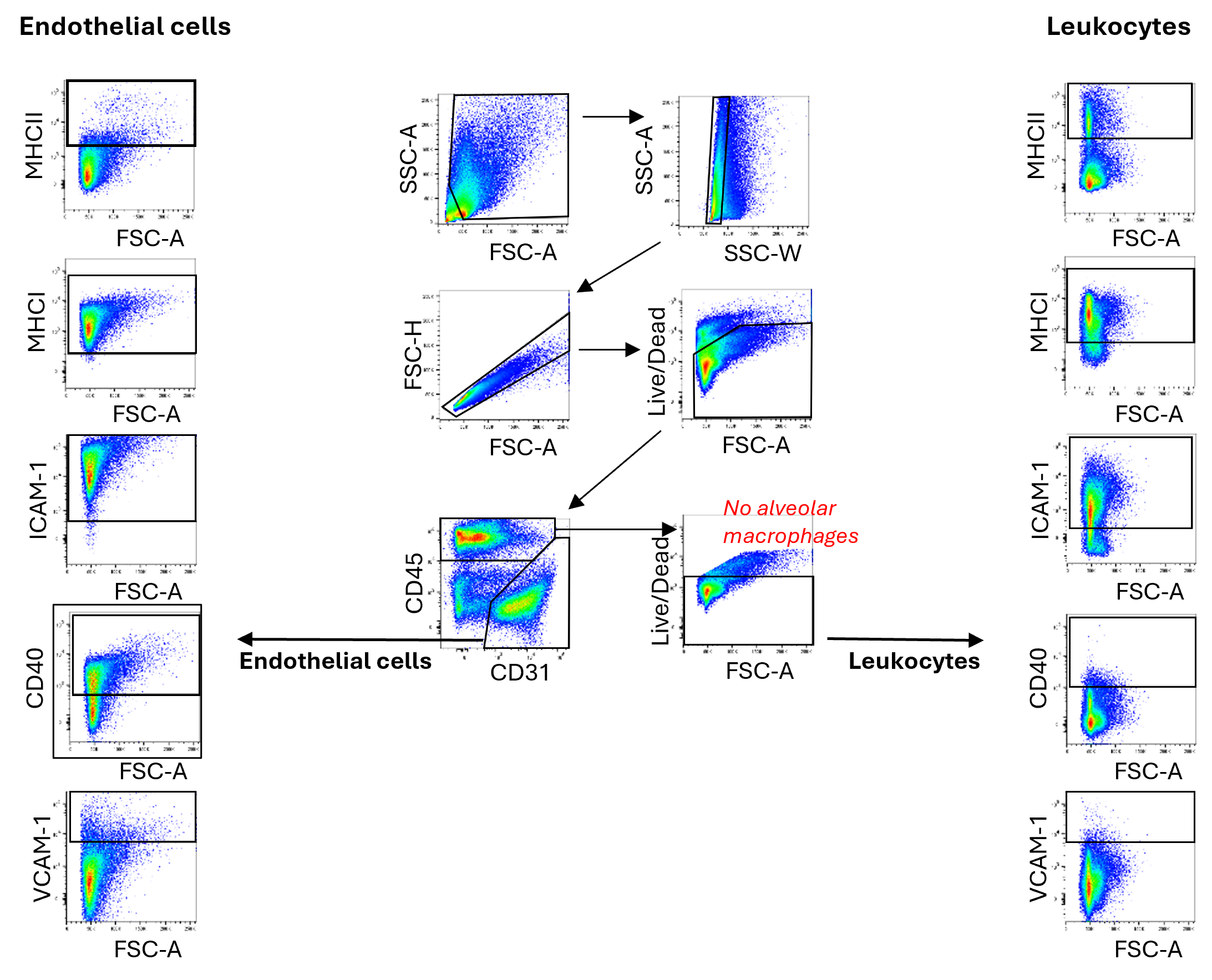

Supplement: Supplementary file 4 [file Image2.tif]

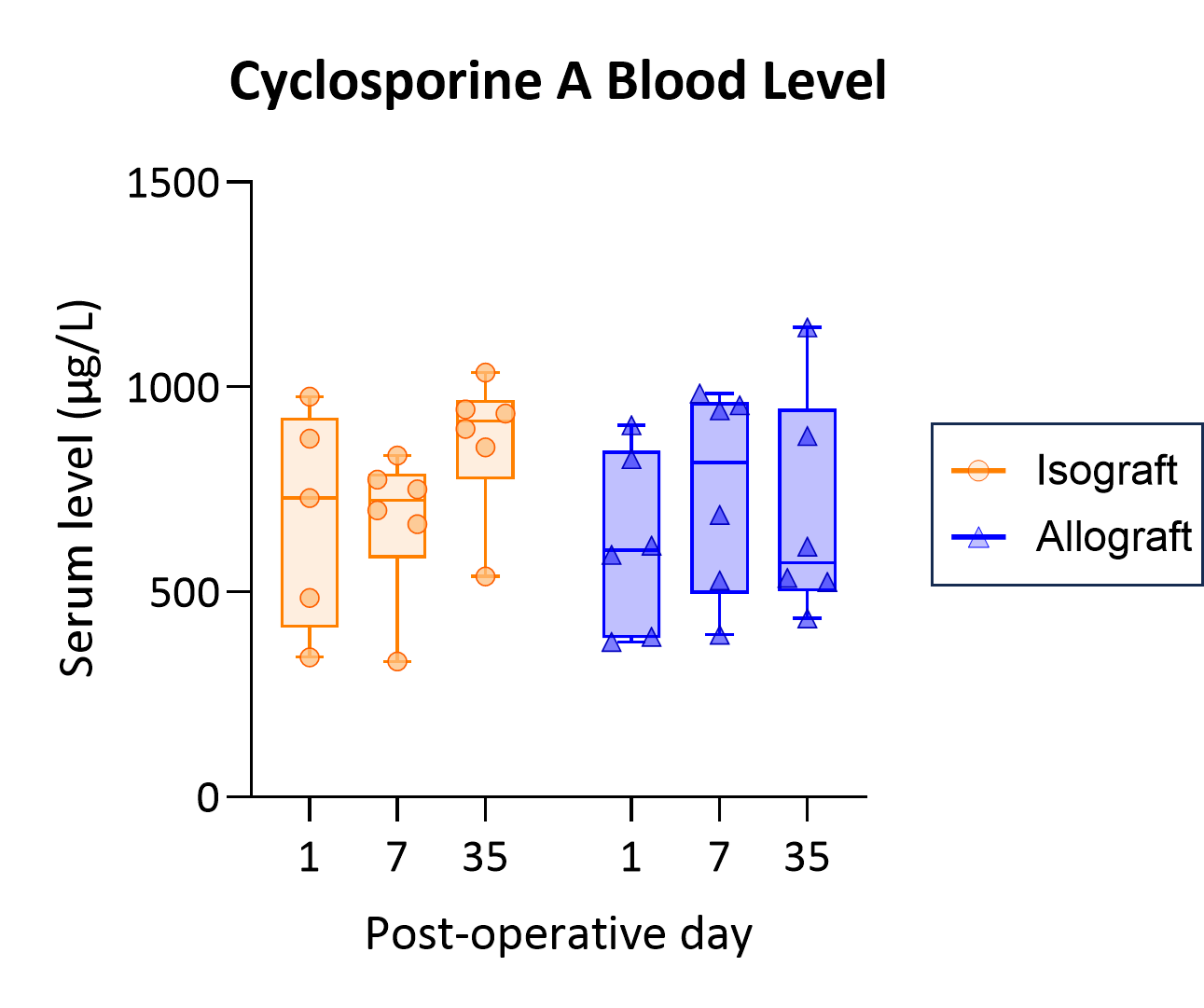

Supplement: Supplementary file 5 [file Image1.tif]
